# Osmoregulation in the Plotosidae Catfish: Role of the Salt Secreting Dendritic Organ

**DOI:** 10.3389/fphys.2018.00761

**Published:** 2018-07-03

**Authors:** Salman Malakpour Kolbadinezhad, João Coimbra, Jonathan M. Wilson

**Affiliations:** ^1^Interdisciplinary Centre of Marine and Environmental Research (CIIMAR/CIMAR), University of Porto, Porto, Portugal; ^2^Instituto de Ciências Biomédicas de Abel Salazar, Universidade do Porto, Porto, Portugal; ^3^Department of Biology, Wilfrid Laurier University, Waterloo, ON, Canada

**Keywords:** osmoregulation, dendritic organ, gill, NKCC1, Na^+^/K^+^-ATPase, *Plotosus lineatus*, CFTR

## Abstract

Unlike other marine teleosts, the Plotosidae catfishes reportedly have an extra-branchial salt secreting dendritic organ (DO). Salinity acclimation [brackishwater (BW) 3aaa, seawater (SWcontrol) 34aaa, and hypersaline water (HSW) 60aaa] for 14 days was used to investigate the osmoregulatory abilities of *Plotosus lineatus* through measurements of blood chemistry, muscle water content (MWC), Na^+^/K^+^-ATPase (NKA) specific activity and ion transporter expression in gills, DO, kidney and intestine. Ion transporter expression was determined using immunoblotting, immunohistochemistry (IHC) and quantitative polymerase chain reaction (qPCR). HSW elevated mortality, plasma osmolality and ions, and hematocrit, and decreased MWC indicating an osmoregulatory challenge. NKA specific activity and protein levels were significantly higher in DO compared to gill, kidney and intestine at all salinities. NKA specific activity increased in kidney and posterior intestine with HSW but only kidney showed correspondingly higher NKA α-subunit protein levels. Since DO mass was greater in HSW, the total amount of DO NKA activity expressed per gram fish was greater indicating higher overall capacity. Gill NKA and V-ATPase protein levels were greater with HSW acclimation but this was not reflected in NKA activity, mRNA or ionocyte abundance. BW acclimation resulted in lower NKA activity in gill, kidney and DO. Cl^-^ levels were better regulated and the resulting strong ion ratio in BW suggests a metabolic acidosis. Elevated DO heat shock protein 70 levels in HSW fish indicate a cellular stress. Strong NKA and NKCC1 (Na^+^:K^+^:2Cl^-^ cotransporter1) co-localization was observed in DO parenchymal cells, which was rare in gill ionocytes. NKCC1 immunoblot expression was only detected in DO, which was highest at HSW. Cystic fibrosis transmembrane regulator Cl^-^ channel (CFTR) localize apically to DO NKA immunoreactive cells. Taken together, the demonstration of high NKA activity in DO coexpressed with NKCC1 and CFTR indicates the presence of the conserved secondary active Cl^-^ secretion mechanism found in other ion transporting epithelia suggesting a convergent evolution with other vertebrate salt secreting organs. However, the significant osmoregulatory challenge of HSW indicates that the DO may be of limited use under more extreme salinity conditions in contrast to the gill based ionoregulatory strategy of marine teleosts.

## Introduction

Osmoregulatory organs in teleost fishes including the gills, kidney and digestive tract are involved in maintenance of body fluid balance (Marshall and Grosell, 2006; Takei and Hwang, 2016). Gills are the first organ to directly sense external osmotic changes that also facilitate either compensatory active uptake (in freshwater) or excretion (in saltwater) of monovalent ions (Na^+^, K^+^, and Cl^-^) to maintain plasma osmolality within a narrow range depending on the environmental salinity (Evans, 2008; Takei and Hwang, 2016). Marine teleosts are hypoosmotic to their environment, which leads to osmotic water loss. They compensate by drinking seawater and absorbing the ingested water by solute (Na^+^ and Cl^-^)-linked water transport in the intestine (see review by Grosell, 2010). In contrast, the role of the intestine is minor in osmoregulation of freshwater fishes, which are hyperosmotic to the environment (Takei and Hwang, 2016). However, ion uptake by the intestine can be substantial in feeding fishes (Wood and Bucking, 2011). The critical role of the marine teleost kidney for divalent ion (Mg^2+^, Ca^2+^, and SO_4_^2-^) secretion has been reported (Beyenbach, 2004; McDonald, 2007), while in freshwater fishes large amounts of dilute urine are produced to compensate for the large water influx via osmosis across the body surfaces (Beyenbach, 2004; Marshall and Grosell, 2006).

In freshwater or seawater, the regulation of the osmolality and ion levels of body fluids of fishes is done actively (Edwards and Marshall, 2012). The plasma osmolalities of euryhaline teleost species of freshwater and marine origin vary, however, in seawater they are maintained at less than 480 mOsm/kg H_2_O (Varsamos et al., 2005). The effects of changing salinity on plasma osmolality and circulating electrolytes have been reported in a number of euryhaline teleosts (e.g., Kang et al., 2008; Sardella et al., 2008; Christensen et al., 2012; Tait et al., 2017). The Plotosidae catfish *Plotosus lineatus* is native to Indo-Pacific coastal waters from Japan to the Red Sea and East Africa and have recently invaded the Mediterranean (Lanzing, 1967; Froese and Pauly, 2018). They are amphidromous and are found in marine and brackishwater (BW), and have also been reported in freshwater (Froese and Pauly, 2018).

The DO is a small fleshy external organ situated on the ventral caudal surface of Plotosidae catfishes, in both sexes from early life stages, that is very close to the urogenital papilla (Hirota, 1895; Lanzing, 1967; Laurenson et al., 1993). The parenchymal cells of the DO form glandular acini that are covered by a stratified squamous epithelium (Van Lennep and Lanzing, 1967; Van Lennep, 1968). Descriptive morphological studies in the gills and DO of *Plotosus lineatus* suggested cellular similarity to fish gill ionocytes and elasmobranch rectal gland cells (Van Lennep and Lanzing, 1967; Van Lennep, 1968; Pucke and Umminger, 1979). The rectal glands from elasmobranchs and specialized salt glands from marine tetrapods (e.g., the nasal salt gland of marine birds, lachrymal gland of marine turtles, and lingual glands in sea snakes, and saltwater crocodiles; Kirschner, 1980; Shuttleworth and Hildebrandt, 1999) have similar parenchymal cell characteristics and the mechanism of NaCl excretion is also similar to the teleost gill chloride cells (secondary activity Cl^-^ secretion) (Silva et al., 1977; Singer et al., 1998; Marshall and Grosell, 2006). The numerous independent origins of these salt glands leads to the hypothesis of a convergent evolution of salt glands across taxa (Babonis and Evans, 2011).

Since the molecular machinery of the osmoregulatory organs (gill, DO, kidney and intestine) in *P. lineatus* are unknown, for the first time in the present study we addressed their molecular mechanisms using a combination of enzymatic analysis, immunohistochemistry, immunoblotting and PCR together with key osmoregulatory indicators (plasma ion levels and osmolality, and muscle water and ion levels) in fish acclimated to different salinities. Salinities covering the natural range of Plotosidae catfishes and a more challenging hypersaline condition that can be present in hypersaline estuaries (Lanzing, 1967; Young and Potter, 2002) were used. In doing so we also addressed the possibly of a conservation of mechanisms for ion transport in salt secretory cells similar to other vertebrate salt glands with respect to co-option events where the original function of the organ is displaced (e.g., lachrymal glands of marine turtles have tear production co-opted for the secretion of concentrated salt solution).

## Materials and Methods

### Animals

Striped eel catfish *Plotosus lineatus* (∼8–13 g; 12.3–14.0 cm; *n* = 54) were obtained from the Tropical Marine Centre (TMC) Portugal and transported to the Laboratory of Molecular Physiology CIIMAR (Porto). Fish were originally collected from the wild in Australian waters for TMC. All fish were acclimatized to laboratory conditions in a 100 L tank of seawater (SW) 34aaa, with mechanical and biological filtration, UV sterilization, aeration, and temperature control (26–28°C) under a natural photoperiod for 3 weeks prior to the start of the experiment to avoid any confounding effects of handling stress on osmoregulation (Biswas et al., 2006). The water salinity, pH, temperature and dissolved oxygen were monitored daily with a multimetric probe (WTW 340i, WTW Measurement Systems Inc., Weilheim, Germany).

### Salinity Acclimation

Three salinity levels were investigated following at least 2 weeks experimental acclimation to BW 3aaa, seawater (SW-control) 34aaa, or hypersaline water (HSW) 60aaa under uniform holding conditions. The salinity extremes were based on preliminary studies. Initially, groups of 5–6 fish were transferred to 30 L tanks, in which salinity was changed in a stepwise fashion, from 34aaa (main tank) to 3 or 60aaa (smaller 30 L tanks), by 5aaa per day. Instant Ocean^®^ sea salts were used to prepare a stock solution of 100aaa and diluted to the appropriate salinities. Aquaria were provided with mechanical and biological filtration, UV sterilization and weekly 20% water changes. The fish were used according to the Portuguese Animal Welfare Law (Decreto-Lei no.197/96) and animal protocols were approved by CIIMAR/UP.

### Sampling

Individual marine catfish were netted then euthanized in a separate smaller tank (1 L) with an overdose of ethyl-m-amino benzoate-MS-222 (1:5000, pH 7.5 adjusted with NaHCO_3_; Pharmaq, United Kingdom), weighed (±0.01 g) and total length (mm) measured. Blood was collected using heparinized capillary tubes following caudal transection then centrifuged at 13000 × *g* for 5 min at room temperature (Heraeus Pico 17 Centrifuge, Thermo Scientific). Hematocrit (Hct) was measured. The isolated plasma was then frozen in liquid nitrogen and kept at -80°C. The following organs were collected: gill, dendritic organ (DO), kidney, and anterior and posterior intestine were snap frozen in liquid nitrogen and were then immediately stored at -80°C. The gastrointestinal tract of *P. lineatus* is relatively short and without a stomach. The anterior intestine was heavily pigmented and easily distinguished from the unpigmented posterior intestine. Gill filaments were sampled from the second arch on the left side, DO, kidney, and intestine were also excised, immersed in 100 μl of ice-cold SEI (150 mM sucrose, 10 mM EDTA, 50 mM imidazole, pH 7.3) buffer and frozen at -80°C. An additional piece of deskinned epaxial muscle (∼1 g) was collected into a pre-weighed tube for water and ion analysis. In additional sets of six individuals, the body cavities were opened and then immersion fixed in 10% neutral buffered formalin (NBF 10%) for 24 h and then stored in 70% ethanol at 4°C.

### Ion Quantification

The ∼1 g of muscle was dried at 60°C to constant mass for the determination of muscle water content (MWC). The dried muscle samples were digested in 65% nitric acid for 3 days at room temperature. The Na^+^ and K^+^ concentrations were quantified by flame photometry (model PFP7; Jenway, Felsted, United Kingdom) and expressed as μmol ⋅ g^-1^ wet mass. Plasma samples were also analyzed by flame photometry (PinAAcle 900T Atomic Absorption Spectrophotometer; Perkin Elmer, Waltham, MA, United States). Chloride concentration was measured in plasma samples colormetrically (Küffer et al., 1975). Plasma osmolality was determined in fresh samples using freezing-point depression osmometry (Micro-Osmometer, Roebling Co., Berlin, Germany) and reported as mOsm kg^-1^ (Malakpour Kolbadinezhad et al., 2012).

### Measurement of Na^+^/K^+^-ATPase Activity

The Na^+^/K^+^-ATPase (NKA) activity was measured according to McCormick (1993) with modifications by Wilson et al. (2007). After thawing samples in 300 μl SEI (250 mM sucrose, 10 mM EDTA, 50 mM imidazole pH 7.3) buffer, sodium deoxycholate was added to a final concentration of 0.1%. Tissue was homogenized using a Precellys 24 bead homogenizer (Bertin Technologies, Montigny-le-Bretonneux, France) at 5800 RPM for 2 × 15 s and then immediately centrifuged at 15,000 × g for 5 min at 4°C. Ten μl of supernatant were run in two duplicate sets for the ATPase assay at 340 nm with a temperature controlled microplate reader (Powerwave 340; Biotek, Winooski, VT, United States) and Gen5^TM^ reader control and data analysis software for 10–20 min at 25°C. One set of wells contained the assay mixture and the other set assay mixture plus ouabain (1 mM, Sigma-Aldrich Chemical Co., St. Louis, MO, United States) to specifically inhibit NKA activity. Total protein was determined in the remaining supernatant using the Bradford (1976) at 600 nm and the results are expressed as μmol ADP mg^-1^ protein h^-1^. In the case of the DO, the NKA activity is also expressed per DO correcting for body mass, since DO mass increase under HSW conditions.

### Immunoblotting

The unused homogenate from the ATPase assay was mixed with an equal volume of 2x Laemmli’s buffer (Laemmli, 1970), heated for 10 min at 70°C and then stored at 4°C. Protein concentration was adjusted to 1 μg μl^-1^ using 1x Laemmli’s buffer. Immunoblotting was performed as described in Reis-Santos et al. (2008). Ponceau-S staining of membranes was performed to assess transfer and sample loading consistency. Blots were probed with heterologous rabbit polyclonal antibodies against the α-subunit of NKA (1:500; αR1 antibody; Wilson et al., 2007), bovine cytosolic CA (1:2000, Abcam, Cambridge, United Kingdom; Randall et al., 2014), and V-ATPase B subunit (1:200, B2; Wilson et al., 2007) or mouse monoclonal antibodies against NKCC/NCC [1:200, T4 clone; Developmental Studies Hybridoma Bank (DSHB), Iowa City, IA, United States; Tipsmark et al., 2002; Wilson et al., 2007], heat shock protein (Hsp70) (1:10000, BRM-22 clone; Sigma-Aldrich), and α-tubulin (1:500 12G10 clone, DSHB). The later antibody was used as a loading control for samples. The membranes were rinsed with TTBS (0.05% Tween-20 in Tris Buffered Saline) and then incubated for 1 h with a goat anti-rabbit or anti-mouse IgG secondary antibodies conjugated to horseradish peroxidase (HRP) and the signal were detected by enhanced chemiluminescence (ECL) using Immobilon Western chemiluminescent HRP substrate (Millipore Corporation, Billerica, MA, United States). Images were acquired using a luminescent image analyzer Fujifilm LAS-4000 mini and image reader software LAS-4000 version.2.0. The area intensity of bands were quantified using the image analysis software program Multi Gauge v3.1 (FUJIFILM, Tokyo, Japan) and expressed as a ratio with α-tubulin.

### Immunohistochemistry (IHC)

Immunofluorescence localization was performed according to Reis-Santos et al. (2008). In short, paraffin serial sections were cut and dewaxed followed by a series of xylene baths and rehydrated through a descending ethanol series to water. Antigen retrieval was performed on some sections (Shi et al., 2011) by pretreatment with 0.05% citraconic anhydride (pH 7.3) for 30 min at 98°C (Namimatsu et al., 2005) and then with 1% sodium dodecyl sulfate (SDS) in PBS for 5 min (Brown et al., 1996). All sections were then blocked with 5% normal goat serum (NGS) and then incubated with primary rabbit or mouse antibodies to the α-subunit of NKA (αR1), NKCC/NCC (T4), CFTR (R&D systems), and V-ATPase B-subunit (B2) overnight at 4°C, rinsed in TPBS (0.05% tween-20 phosphate buffered saline, pH 7.4), followed by incubation with secondary goat anti-mouse Alexa Fluor 568 and/or goat anti-rabbit Alexa Fluor 488-conjugated antibodies for 1 h at 37°C. Sections were stained with DAPI (4′,6-diamidino-2phenylindole) and viewed on a Leica DM6000B wide field epifluorescence microscope and micrographs taken with a digital camera (DFC340FX, Leica Microsystems, Wetzlar, Germany) using Leica LAS AF acquisition software. Figures were assembled in Photoshop CS3.

### Molecular Genetics Approach

#### Isolation and Quantification of RNA and Synthesis of Complementary DNA

Frozen pieces of gill, dendritic organ, kidney, and intestine were thawed in lysis buffer and homogenized using a Precellys 24 bead homogenizer (6400 RPM 2 × 10 s; Bertin) and RNA isolated using silica-based columns (Aurum Total RNA mini kit, Bio-Rad) according to the manufacturer’s instructions. Total RNA concentration and purity were determined using a Nanodrop spectrophotometer (Thermo Scientific, Wilmington, DE, United States). The isolated total RNA samples were then stored at -80°C. For each sample 1 μg of total RNA was converted to cDNA using an iScript cDNA kit (Bio-Rad) according to the manufacturer’s instructions. Samples were stored at -20°C.

#### Gene Isolation

Consensus primers were designed from a conserved region of β-actin (*actb*, *Sparus aurata*, Santos et al., 1997), Na^+^/K^+^-ATPase α-subunit (*atp1a*, *Anguilla anguilla*, Cutler et al., 1995), cystic fibrosis transmembrane conductance regulator [*cftr* (abcc7) *Ictalurus punctatus*, Liu et al., 2016], cytosolic carbonic anhydrase (*ca17*; *Danio rerio*, Ferreira-Martins et al., 2016), putative anion transporter Cl^-^/HCO_3_^-^ exchanger (*slc26a6*, *D. rerio*, *Tetraodon nigroviridis*, *A. anguilla*, *Xenopus laevis*, *Homo sapiens*, Grosell et al., 2009b) by multiple sequence alignment (Corpet, 1988). Nucleotide sequences and amplicon sizes of these primers are shown in **Supplementary Table S1**. PCR products were cloned (pGEM-T Easy Vector System, Promega, Madison, WI, United States), sequenced (StabVida, Oeiras, Portugal) and identity confirmed using bioinformatic tools (BLAST, ClustalX). The specific primer for *P. lineatus* were designed with Primer3 (Rozen and Skaletsky, 2000) and initially tested for specificity by RT-PCR (**Supplementary Tables S2**). Primer sequences and amplicon sizes are shown in **Supplementary Table S1**.

#### RT-PCR and Real-Time PCR

The RT-PCRs were performed using GoTaq^®^ DNA polymerase (Promega) and Phusion Flash (Thermo Fisher Scientific) for actin and other interested genes, respectively.

The real-time quantitative PCR (qPCR) was performed using SYBR green with an iQ5 Multicolor Real-Time PCR Detection System (Bio-Rad) (**Supplementary Table S2**). Reaction efficiency was determined by performing qPCR reactions with a pooled cDNA dilution series for each gene of interest. β-Actin was used as a reference gene. The expression levels of the genes of interest were analyzed based on cycle threshold (CT) values using the comparative CT method (2^-ΔΔCT^) (**Supplementary Table S3**) (Livak and Schmittgen, 2001). A melt curve for every PCR product was generated to confirm the specificity of the assays. PCR products were cloned (pGEM-T easy vector system, Promega) and sequenced to confirm primer specificity (StabVida).

### Statistics

Data are presented as means ± standard error of the mean (SEM). Statistical differences of protein, and mRNA expression between groups were determined using one-way analysis of variance (ANOVA) followed by the *post hoc* Student–Newman–Keuls (SNK) test (SigmaPlot 11.0 Systat Software, Inc.) in juveniles exposed to different salinities. Data were square root or log transformed in the case of a failed normality test. The fiducial limit was set at 0.05.

## Results

### Osmoregulatory Indicators

Plasma and muscle osmoregulatory indicators are presented in **Table 1**. Plasma Na^+^ concentrations correlated positively across the range of acclimation salinity while plasma Cl^-^ and Ca^2+^ concentrations and osmolality were significantly higher in HSW compared with SW and BW acclimated animals. Plasma osmolality was more than 50% higher in HSW acclimated fish. The resulting plasma strong ion ratio (SIR) was significantly lower in BW fish compared to SW and HSW acclimation. Hematocrit showed a positive correlation with salinity where BW values were half of HSW. Acclimation salinity had no effect on plasma K^+^ concentration.

**Table 1:** Fish morphometrics: condition factor (K) and DO mass (mg DO per g body mass); plasma Na^+^, Cl^-^, K^+^, and Ca^2+^ concentrations and osmolality, hematocrit, strong ion ratio (SIR; Na^+^:Cl^-^) and muscle water content (MWC%), Na^+^ and K^+^ concentrations, and Na^+^/K^+^ ratio of *P. lineatus* acclimated to [brackish water (BW) 3aaa, seawater (SW control) 34aaa, and hypersaline water (HSW) 60aaa].

**Morphometrics**			
Condition Factor (K)	5.75 ± 0.17^a^	4.60 ± 0.15^b^	4.17 ± 0.10^c^
mg DO /g body mass	1.25 ± 0.06^a^	0.90 ± 0.04^b^	2.65 ± 0.10^c^
**Plasma**	**BW (3aaa)**	**SW Control (34aaa)**	**HSW (60aaa)**
Na^+^(mmol l^-1^)	119.8 ± 5.4^a^	152.6 ± 5.6^b^	186.2 ± 16.7^c^
Cl^-^ (mmol l^-1^)	125.5 ± 4.2 ^a^	127.8 ± 3.9^a^	148.7 ± 8.0^b^
K^+^(mmol l^-1^)	4.6 ± 0.5	5.1 ± 0.4	5.1 ± 0.3
Ca^2+^ (mmol l^-1^)	2.6 ± 0.2^a^	3.0 ± 0.2^a^	3.8 ± 0.5^b^
Osmolality(mOsm)	391.2 ± 25.1^a^	374.4 ± 26.0^a^	588.0 ± 48.7^b^
Hematocrit (%)	15.3 ± 1.2^a^	23.3 ± 2.8^b^	29.2 ± 1.7^c^
SIR (Na^+^:Cl^-^ ratio)	0.96 ± 0.04^a^	1.20 ± 0.06^b^	1.24 ± 0.09^b^
**Muscle**			
MWC (%)	86.6 ± 1.7^a^	87.5 ± 1.6^a^	77.4 ± 0.3^b^
Na^+^(mmol kg^-1^)	66.9 ± 8.9	64.2 ± 4.2	67.4 ± 5.0
K^+^(mmol kg^-1^)	138.5 ± 9.6^a^	138.6 ± 2.4^a^	204.0 ± 8.0^b^
Na^+^:K^+^ ratio	0.48 ± 0.92^a^	0.46 ± 1.73^a^	0.33 ± 0.63^b^

*Data are means ± SEM. (*n* = 7–9). Salinity difference within a given parameter that do not share the same letter(s) are significantly different from one another*.

Muscle water content was significantly lower in HSW acclimated fish indicating dehydration but was unaffected by BW acclimation. In contrast, fish condition factor showed a negative correlation with salinity from BW to HSW. Muscle K^+^ concentration followed the opposite trend being significantly higher in HSW fish. Muscle Na^+^ content did not differ with salinity, which was reflected in a lower Na^+^: K^+^ ratio in HSW fish. There was mortality (36%) only with HSW acclimation but not in other salinity groups.

### NKA Activity

**FIGURE 1:**
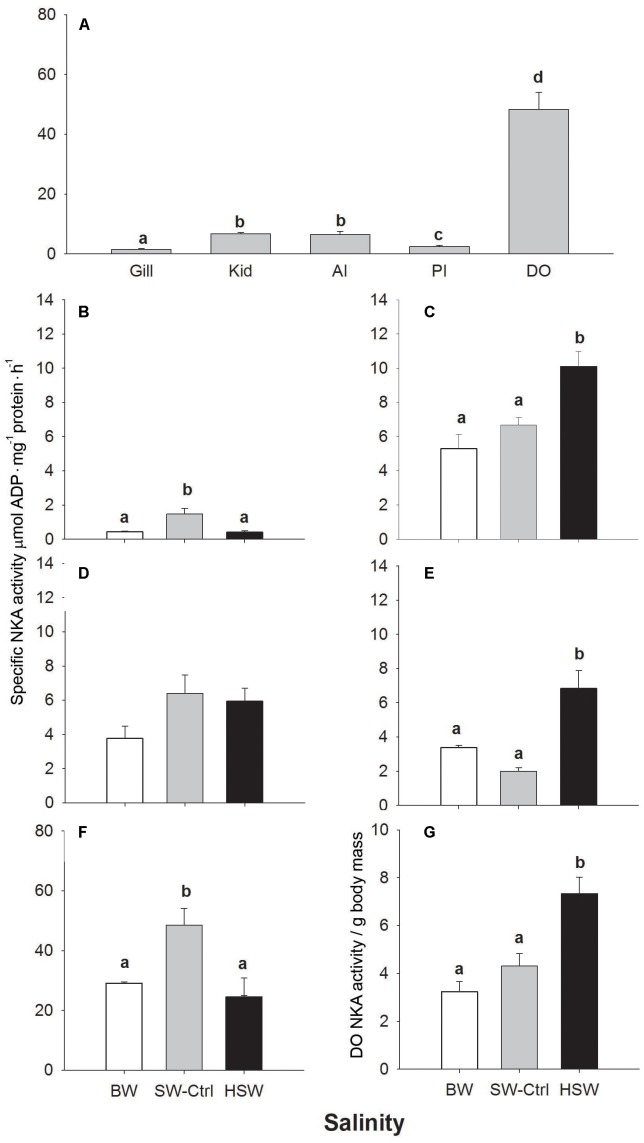
Na^+^/K^+^-ATP (NKA) activity in different osmoregulatory organs of SW acclimated *P. lineatus* **(A)**. Organ specific NKA activity [gill **(B)**, kidney **(C)**, anterior **(D)** and posterior intestines **(E)**, and dendritic organ **(F)**] in *P. lineatus* acclimated to brackishwater (BW) 3aaa, seawater (SW control) 34aaa, and hypersaline water (HSW) 60aaa. Total DO NKA activity per g body mass (G). Values are means ± SEM. (*n* = 5–6). Different lower case letters indicate a significant difference between organs **(A)** and with salinity within each tissue **(A–G)** (*P* < 0.05).

In SW *P. lineatus*, the specific NKA activity was lowest in the gill and posterior intestine, and more than three times higher in the kidney and anterior intestine and twenty times higher in the DO (**Figure 1A**). In response to salinity acclimation, similar patterns of NKA specific activity were detected in the gill and DO with significantly higher activity in SW acclimated fish compare to both BW and HSW salinities (**Figures 1B,F**). In both the kidney and posterior intestine, NKA activity was significantly higher in HSW, with no differences between SW and BW (**Figures 1C,E**). Acclimation to different salinities did not affect the NKA activity in the anterior intestine (**Figure 1D**).

The mass of the DO in SW-control salinity acclimated fish expressed as a percentage of fish body mass was significantly lower compared to BW and HSW salinity groups (**Table 1**). However, in HSW salinity acclimated fish DO mass was greatest at 213 and 243% of BW and SW fish, respectively. Since DO mass changed with salinity, we also expressed NKA activity on a whole organ basis corrected for fish mass. The expression of the total DO NKA activity relative to fish body mass showed that in HSW fish DO NKA activity was 1.6 and 2.1 fold higher than in SW and BW fishes, respectively (**Figure 1G**).

### Immunoblotting

We used antibodies crossreactive with NKA α-subunit, NKCC1, cytosolic carbonic anhydrase (Ca17), V-ATPase B subunit and Hsp70 to determine how salinity affected the abundance of these important transport and stress related proteins in key osmoregulatory organs: gill, kidney, DO, and anterior and posterior intestines. A representative tissue distribution immunoblot is shown in **Supplementary Figure S1** of a SW control fish.

**FIGURE 2:**
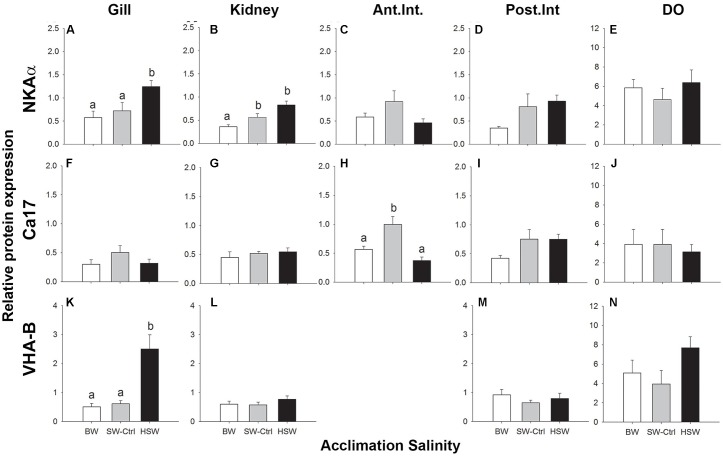
Immunoblotting relative expression of NKA α-subunit (αR1 antibody; **A–E**), cytosolic carbonic anhydrase (Ca17; **F–J**) and V-ATPase B subunit (B2 antibody; **K–N**) in the gill **(A,F,K)**, kidney **(B,G,L)**, anterior and posterior intestines **(C,D,H,I,M)** and DO **(E,J,N)** *P. lineatus* were acclimated to [BW 3aaa, seawater (SW control) 34aaa, and HSW 60aaa]. α tubulin (12G10 antibody) was used as a loading control. Values are presented as means ± SEM of protein abundance (*n* = 5–6). Different letters indicate a significant difference between salinities, one-way analysis of variance (ANOVA) and SNK (*P* < 0.05).

**FIGURE 3:**
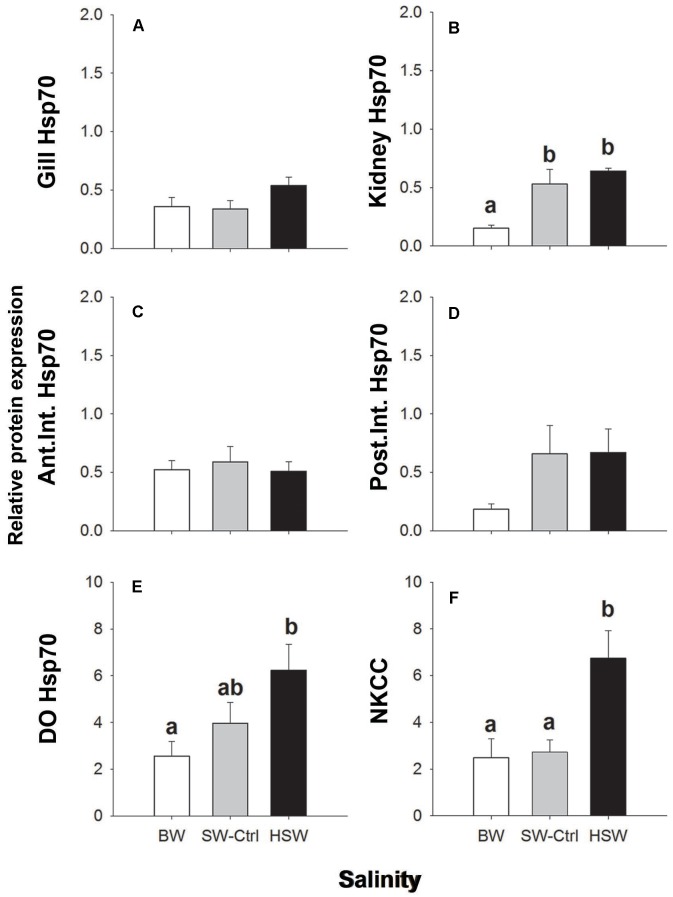
Immunoblotting relative expression of heat shock protein 70 (Hsp70) in the gill **(A)**, kidney **(B)**, anterior and posterior intestines **(C,D)** and DO **(E)** and NKCC in dendritic organ **(F)** of *P. lineatus* acclimated to [BW 3aaa, seawater (SW control) 34aaa, HSW 60aaa]. α tubulin (12G10 antibody) was used as a loading control. Values are means ± SEM (*n* = 5–6). Different letters indicate a significant difference between salinities, one-way ANOVA and SNK (*P* < 0.05).

NKA α-subunit expression was detected in all organs of interest as a single band of approximately 100 kDa. The relative expression of the NKA α-subunit protein was significantly higher with HSW exposure in the gill and kidney (**Figures 2A,B**) but was not salinity responsive in either the intestine or DO (**Figures 2C–E**). NKCC/NCC expression was detected only in DO with a pair of prominent immunoreactive bands of 140–260 kDa with some additional higher molecular mass bands sometimes present. Higher NKCC/NCC expression in the HSW salinity acclimated fish relative to BW and SW fish in DO was observed (**Figure 3F** and **Supplementary Figure S1**). The expression intensities of these bands were approximately 2.7 and 2.5 time greater in HSW acclimated individuals compare to the BW and SW-controls, respectively. Immunoreactivity with the CFTR antibody was observed in the predicted molecular mass range as a single band of 160 kDa in DO of a SW fish (**Supplementary Figure S1**); however, blots for the salinity experiment were not clean, and multiple smaller cross-reactive bands were detected, which made semi-quantification problematic. Because of these difficulties in detecting cross-reactive bands, the antibody was not use in other organs for quantification.

Ca17 was detected as an approximately 30 kDa band in all organs (**Figures 2F–J** and **Supplementary Figure S1**). Relative Ca17 protein expression was significantly lower with HSW in anterior intestine relative to the SW control and BW fish. No detectable differences were found in the DO, kidney or posterior intestine. The V-ATPase B subunit was expressed as a ∼56 kDa band in the gill, kidney, DO and posterior intestine but not anterior intestine (**Figures 2K–N** and **Supplementary Figure S1**). The relative protein expression in the gill was highest in HSW compared to BW and SW. No detectable differences were found in the kidney, posterior intestine or DO with salinity acclimations.

Hsp70 protein was found in all of the organs of interest as a single 70 kDa immunoreactive band (**Figures 3A–E**). Hsp70 showed significantly higher levels with HSW in the DO and kidney relative to BW with intermediate levels in SW. No other differences with salinity were observed in gill or intestine.

### Gene Expression of *atp1a1,* *ca17*, *cftr*, *slc26a6a*

Using a PCR based approach we identified orthologs of *atp1a1*, *cftr* (*abcc7*), *ca17*, and *slc26a6* in *P. lineatus* from partial sequences. Percentage amino acid identities for each gene compared to channel catfish (*Ictalurus punctatus*), rainbow trout (*O. mykiss*) and zebrafish (*D. rerio*) show a high degree of similarity (**Supplementary Table S3**). A phylogentic tree constructed using the Neighbor-Joining method for *P. lineatus* Atp1a1 shows that it is found in the Atp1a1 clade (**Supplementary Figure S2**).

Significant effects were seen in NKA α-subunit *atp1a1* mRNA expression levels in DO with a negative correlation with salinity (higher expression in BW compared to HSW) (**Table 2**). No salinity dependent effects were observed in any other tissue.

**Table 2:** Relative mRNA expression of *atp1a1*, Na^+^/K^+^-ATPase; *cftr*, cystic fibrosis transmembrane conductance regulator; *ca17*, cytosolic carbonic anhydrase; and *slc26a6*, putative anion transporter Cl^-^/HCO_3_^-^ exchanger genes in the gill, DO, kidney, anterior and posterior intestines of marine catfish *P. lineatus* acclimated to [brackishwater (BW) 3aaa, seawater (SW-control) 34aaa, hypersaline water (HSW) 60aaa].

Organ	Genes	BW (3aaa)	SW (34aaa)	HSW (60aaa)
Gill	*atp1a1*	1.61 ± 0.40 (4)	1.00 ± 0.13 (3)	0.66 ± 0.06 (3)
	*cftr*	0.97 ± 0.48	1.00 ± 0.38	0.50 ± 0.10
	*ca17*	1.58 ± 0.08^a^	1.00 ± 0.13^b^	1.19 ± 0.21^ab^
	*slc26a6*	1.04 ± 0.65	1.00 ± 0.33	1.47 ± 0.72
Kidney	*atp1a1*	1.57 ± 0.45 (4)	1.00 ± 0.47 (3)	1.62 ± 0.56 (3)
	*cftr*	14.02 ± 6.42^a^	1.00 ± 0.46^b^	0.42 ± 0.18^b^
	*ca17*	1.60 ± 0.43^a^	1.00 ± 0.35^a^	3.00 ± 0.22^b^
	*slc26a6*	6.19 ± 2.21^a^	1.00 ± 0.25^b^	1.89 ± 1.15^b^
Ant int	*atp1a1*	0.84 ± 0.06 (3)	1.00 ± 0.37 (3)	0.77 ± 0.17 (3)
	*cftr*	1.45 ± 0.28^ab^	1.00 ± 0.33^a^	2.93 ± 0.71^b^
	*ca17*	0.70 ± 0.22	1.00 ± 0.38	0.88 ± 0.14
	*slc26a6*	1.13 ± 0.10	1.00 ± 0.48	1.46 ± 0.21
Post int	*atp1a1*	1.44 ± 0.32 (4)	1.00 ± 0.23 (3)	1.71 ± 0.08 (3)
	*cftr*	0.78 ± 0.22	1.00 ± 0.14	0.84 ± 0.58
	*ca17*	0.77 ± 0.13	1.00 ± 0.35	0.81 ± 0.32
	*slc26a6*	0.95 ± 0.39	1.00 ± 0.08	1.20 ± 0.50
DO	*atp1a1*	1.60 ± 0.36^a^ (5)	1.00 ± 0.11^ab^ (4)	0.65 ± 0.06^b^ (3)
	*cftr*	1.37 ± 0.17^a^	1.00 ± 0.06^a^	0.30 ± 0.05^b^
	*ca17*	0.65 ± 0.07^a^	1.00 ± 0.10^b^	0.44 ± 0.05^c^
	*slc26a6*	1.47 ± 0.38^a^	1.00 ± 0.15^b^	15.12 ± 1.67^c^

*Data are means ± SEM. Sample sized are indicated in brackets (n) for each organ. The amounts of mRNAs are normalized to the corresponding *bact* abundance from the same sample and the expressed relative to the SW control group. Different letters indicate a significant difference between salinities, one-way analysis of variance (ANOVA) (*P* < 0.05; see text for details)*.

The HSW acclimated fish had higher *cftr* mRNA expression in the anterior intestine but lower in the DO relative to SW fish (**Table 2**). In the kidney, BW acclimation was associated with significantly higher mRNA levels whereas in all other organs BW was not associated with any significant difference from SW. There were no salinity dependent effects in the gill or posterior intestine.

The *ca17* mRNA expression showed higher levels with HSW in the kidney in contrast to lower levels in the DO (**Table 2**) compared to BW and SW-controls. In BW the gill showed higher expression than the SW-control but was not different from HSW fish. However, in the DO of BW fish expression was lower than the SW-control but the lowest expression was found in the HSW fish. There were no salinity dependent effects in either the anterior or posterior intestines.

The *slc26a6a* was expressed in all organs studied. Only in the DO did HSW acclimation show higher *slc26a6a* mRNA expression, whereas in the kidney BW acclimation resulted in higher expression (**Table 2**). There were no other salinity dependent effects in the gills or intestine.

### Immunohistochemistry

#### Gill

**FIGURE 4:**
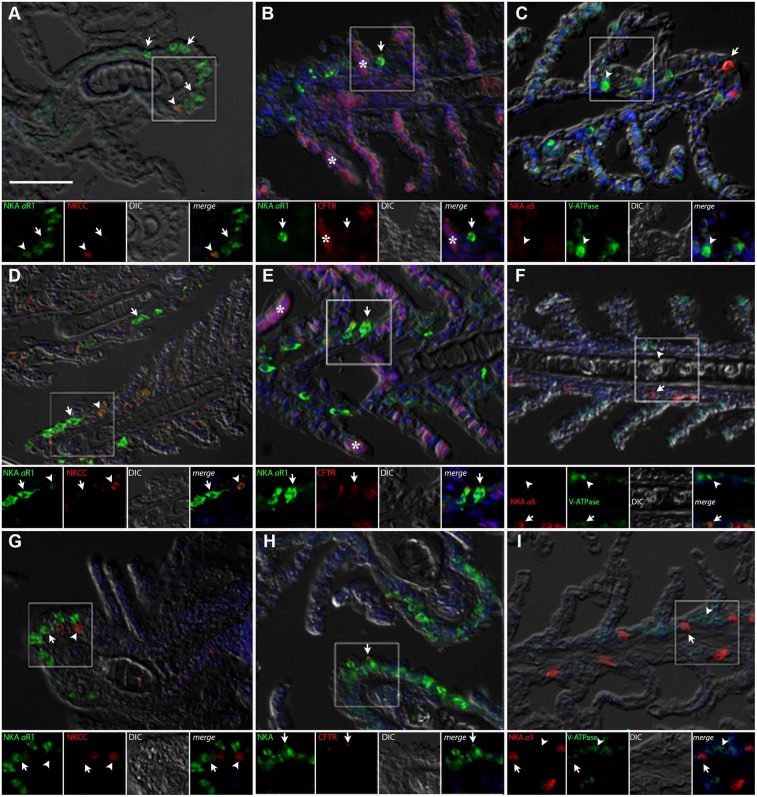
Immunofluorescence localization of Na^+^/K^+^-ATPase (αR1, green **A,B,D,E,G,H**) with NKCC1 (T4, red **A,D,G**) and CFTR (red, **B,E,H**) or Na^+^/K^+^-ATPase (α5, red **C,F,I**) with V-ATPase (B2, green **C,F,I**) in the gills of *P. lineatus*. Acclimation was performed in brackish water (BW) 3aaa **(A–C)**, seawater (SW control) 34aaa **(D–F)** and HSW 60aaa **(G–I)**. Sections were counter stained with DAPI nuclear staining (blue) and overlaid with the differential interference contrast (DIC) images. Insets show the separate channels of the framed area in the respective panels **(A–I)**. In **(A,D,G)** arrowheads indicate NKCC1 IR cells and arrows NKA-IR cells. In **(B,E,H)** arrows indicate CFTR + NKA IR cells, asterisks red blood cell non-specific fluorescence. In **(C,F,I)** arrowheads indicate V-ATPase IR cells and arrows NKA IR cells. Scale bar 100 μm.

The gills of *P. lineatus* have a typical teleost gill organization of filaments with lamellae. In the branchial epithelium strong NKA immunoreactivity (IR) was detected with both α5 and αR1 antibodies in large isolated ovoid cells throughout the cytoplasm with the exception of the apical region (**Figures 4A–I**). This NKA cellular staining pattern is typical of teleost fish chloride cell or ionocyte tubular system, which is continuous with the basolateral membrane. There were relatively few of these branchial NKA-IR cells which were present in a heterogeneous distribution limited to a few interlamellar regions over the leading edge of the filament and were absent from the lamella. Experimental salinities did not alter the NKA-IR cell distribution pattern. The secretory Na^+^:K^+^:2Cl^-^ cotransporter (NKCC1) expression in gill was rarely detected despite the use of antigen retrieval techniques and positive immunoreactivity in other organs (DO, kidney and intestine) indicating that species specific immunoreactivity problems were not an issue. The colocalization of NKCC1 in more weakly NKA-IR cells in BW and SW fish are shown in **Figure 4A,D**, respectively. Ovoid cells deeper within the filament epithelium showing only NKCC1 staining were observed in HSW (**Figure 4G**).The apical localization of CFTR was detected in some NKA-IR cells with no apparent salinity dependent differences (**Figures 4B,E,H**). The V-ATPase H^+^-pump was localized in a similar cytoplasmic staining pattern as NKA; however, in separate cells from NKA-IR cells under all acclimation conditions (**Figures 4C,F,I**).

#### Dendritic Organ

**FIGURE 5:**
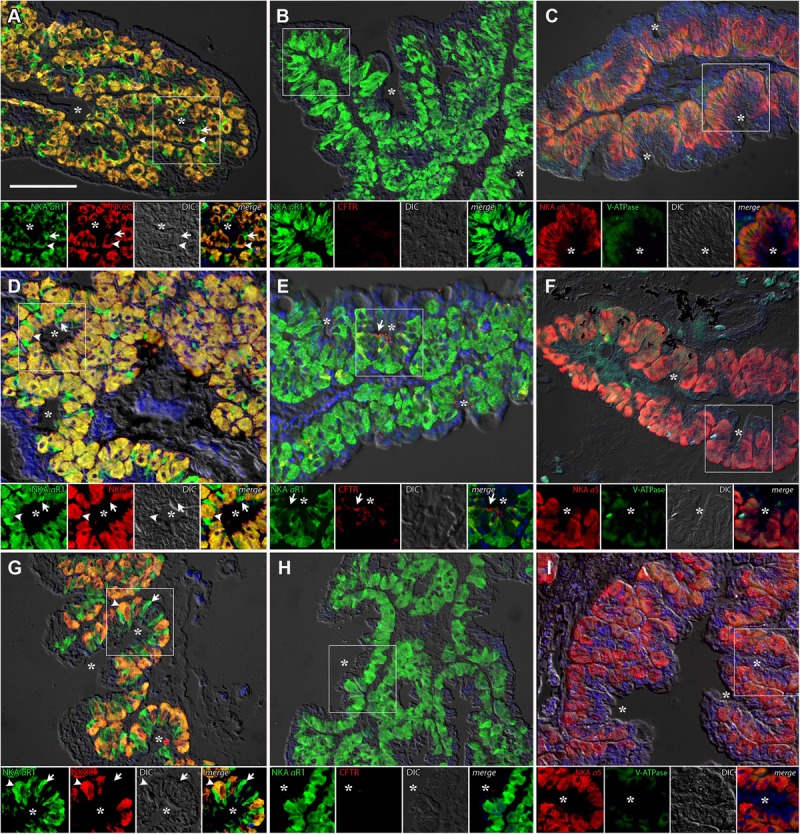
Immunofluorescence localization of Na^+^/K^+^-ATPase (αR1, green **A,B,D,E,G,H**) with NKCC1 (T4, red **A,D,G**) and CFTR (red **B,E,H**) or Na^+^/K^+^-ATPase (α5, red **C,F,I**) with V-ATPase (B2, green **C,F,I**) in the DO of *P. lineatus*. The *P. lineatus* acclimated in brackish water (BW) 3aaa **(A–C)**, seawater (SW control) 34aaa **(D–F)** and HSW 60aaa **(G–I)**. In **(A,D,G)** arrowheads indicate NKCC1 + NKA IR cells and arrow NKA only IR cells. In **(B,E,H)** arrows indicate CFTR + NKA IR cells. Asterisks (^∗^) indicate water. See **Figure 4** caption for additional information. Scale bar 100 μm.

The DO of *P. lineatus* are external and have branching irregular lobes that are well vascularized. The large parenchynal cells form acini covered by a squamous stratified layer of epithelial cells. The large ovoid to pear-shaped parenchymal cells of the DO generally showed strong NKA and NKCC1 immunoreactivity throughout the cell indicative of basolateral tubular system staining (**Figures 5A,D,G**). However, there is a smaller subpopulation of parenchymal cells that are more angular in shape that have noticeably stronger NKA-IR and lack NKCC-IR. Salinity dependent differences in staining were not observed. The apical chloride channel CFTR was only observed in a SW control fish and was generally not detectable despite the use of antigen retrieval techniques and positive immunoreactivity in other organs (gill) indicating that species specific immunoreactivity problems were not an issue (**Figures 5B,E,H**). V-ATPase-IR showed rather similar cytosolic localization in parenchymal cells of the DO ionocytes without salinity dependent differences (**Figures 5C,F,I**).

#### Intestine

**FIGURE 6:**
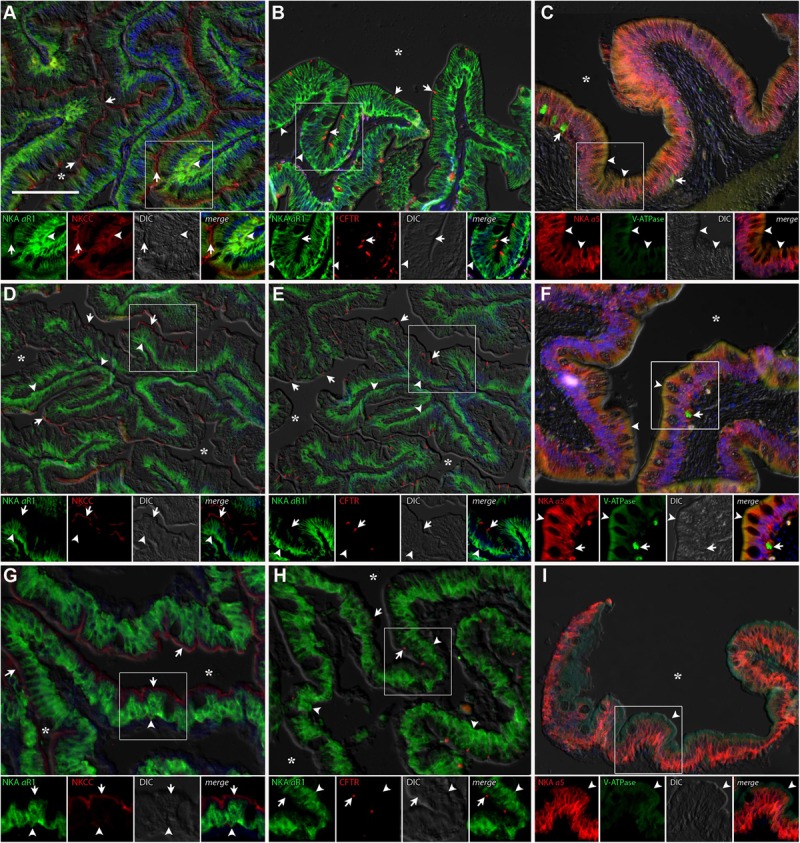
Immunofluorescence localization of Na^+^/K^+^-ATPase (αR1, green **A,B,D,E,G,H**) with NKCC2/NCC (T4, red **A,D,G**) and CFTR (red, **B,E,H**) or Na^+^/K^+^-ATPase (α5, red **C,F,I**) with V-ATPase (B2, green **C,F,I**) in the anterior intestine of *P. lineatus*. The acclimation was performed in brackish water (BW) 3aaa **(A–C)**, seawater (SW control) 34aaa **(D–F)** and HSW 60aaa **(G–I)**. In **(A,D,G)** arrows indicate apical NKCC2/NCC IR cells and arrowheads basolateral NKA-IR [and NKCC1 in **(A)** only]. In **(B,E,H)** arrows indicate CFTR and arrowheads basolateral NKA IR. In **(C,F,I)** arrowheads indicate subapical V-ATPase-IR and arrows basal cells V-ATPase-IR. Asterisks (^∗^) indicate intestinal lumen. See **Figure 4** caption for additional information. Scale bar 100 μm.

**FIGURE 7:**
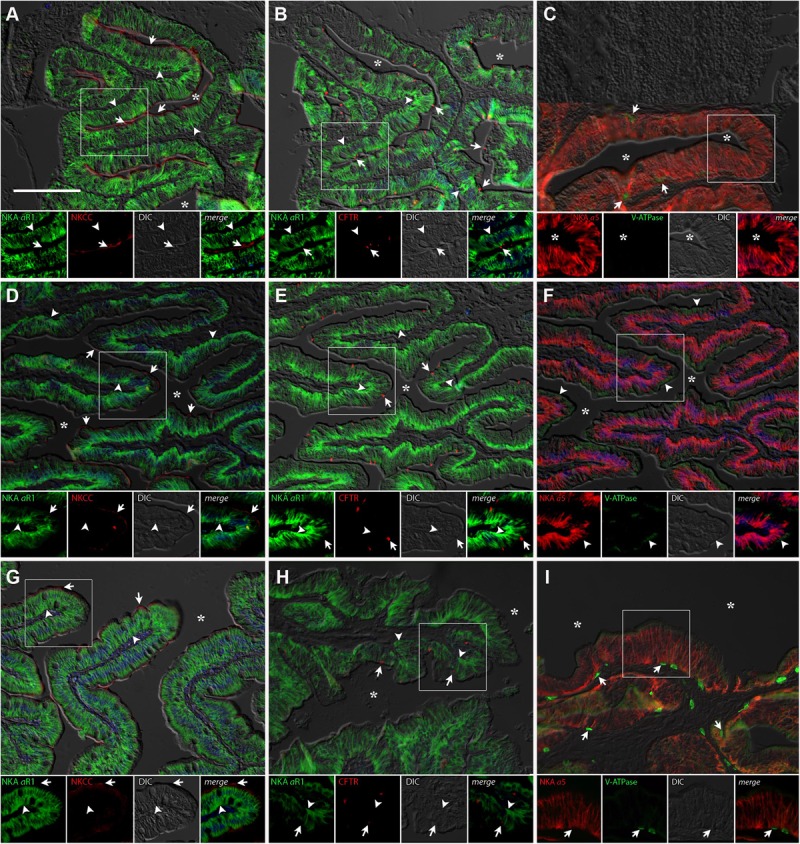
Immunofluorescence localization of Na^+^/K^+^-ATPase (αR1, green **A,B,D,E,G,H**) with NKCC2/NCC (T4, red **A,D,G**) and CFTR (red, **B,E,H**) or Na^+^/K^+^-ATPase (α5, red **C,F,I**) with V-ATPase (B2, green **C,F,I**) in the posterior intestine of *P. lineatus*. Marine catfish acclimated to BW 3aaa **(A–C)**, seawater (SW control) 34aaa **(D–F)** and HSW 60aaa **(G–I)**. In **(A,D,G)** arrows indicate apical NKCC2/NCC IR cells and arrowheads basolateral NKA-IR. In **(B,E,H)** arrows indicate CFTR and arrowheads basolateral NKA IR. In **(C,F,I)** arrowheads indicate subapical V-ATPase-IR and arrows basal cells V-ATPase-IR. Asterisks (^∗^) indicate intestinal lumen. See **Figure 4** caption for additional information. Scale bar 100 μm.

Immunolabelling of NKA in the anterior and posterior intestines of *P. lineatus* acclimated to BW, SW-control or HSW revealed intense staining in the basolateral regions of the intestinal epithelium (**Figures 6**, **7**). NKCC2 or NCC immunoreactivity was detected in apical brush border of the epithelium in the anterior and posterior intestines in all salinity experiments (**Figures 6**, **7A,D,G**). However, basal staining was also detected in anterior intestine of BW fish (**Figure 6A**). CFTR immunoreactivity was detected apically in isolated spindle shaped columnar cells in the epithelium of the anterior and posterior intestines in all of salinity experiment (**Figures 6**, **7B,E,H**).

In the anterior intestine, V-ATPase was found weakly staining the subapical region of columnar epithelial cells at all salinities although much more weakly in HSW (**Figures 6C,F,I**). Staining was not observed in the brush border. Stronger staining is also observed in isolated basal cells in the epithelium and in the lamina propria. In the posterior intestine, subapical expression of V-ATPase in SW has been observed, while in HSW fish basal epithelial cells and cells in the lamina propria show strong immunoreactivity (**Figures 7F,I**). In BW fish, V-ATPase-IR was detected in basal epithelial cells (**Figure 7C**).

## Discussion

*Plotosus lineatus* can osmoregulate across a wide range of salinities (3–34aaa) although HSW (60aaa) conditions presented a significant challenge. BW-SW represents the more natural salinity range of Plotosidae catfishes while HSW would only be encountered in closed or inverted estuaries (Lanzing, 1967; Young and Potter, 2002). However, HSW acclimation has allowed us to test the osmoregulatory abilities of *P. lineatus* under more challenging conditions (Gonzalez, 2012). The DO of *P. lineatus* has the molecular machinery for active NaCl secretion using the conserved mechanism of secondary activity Cl^-^ transport with NKA, NKCC1 and likely CFTR at its core. The gill clearly has a secondary role in ion regulation with few ionocytes and low overall NKA expression. The intestine shows typical attributes of marine teleosts.

### Osmo- and Iono-Regulatory Responses to Salinity Acclimation

The observed plasma ion concentrations were in the range of other teleost fish species (see reviews by Freire and Prodocimo, 2007; Whittamore, 2012). However, in comparison to other studies in Plotosidae, the plasma Na^+^, Cl^-^, and K^+^ concentrations of *P. lineatus* in SW controls in the present study were less than those of *P. lineatus* studied by Pucke and Umminger (1979), while Na^+^ was not very different from *Cnidoglanis macrocephalus* (Kowarsky, 1973). In both of these studies osmolality was also lower. These observed differences might be due to a number of differences between the studies (sampling and analytical methods, acclimation temperatures 26–28°C vs. 19–20°C, species differences). Salinity challenges typically alter plasma osmolality and electrolytes levels in euryhaline teleosts with an initial crisis stage followed by a regulatory stage (Madsen and Naamansen, 1989; Jensen et al., 1998; Wang et al., 2009). *Plotosus lineatus* acclimated to HSW had higher plasma osmolality and ions (except K^+^), and hematocrit, and decreased MWC and condition factor. Together, these data indicate a systemic dehydration due to water loss by osmosis, and elevated plasma osmolality representing disturbances from an internal fluid shift, which may be problematic resulting in a stress situation and mortality. Thus long-term survival in HSW is likely limited. As a corollary, the Plotosidae catfish *C. macrocephalus* in the closed hypersaline Wellstead Estuary (Australia) was not found in the most hypersaline areas of the estuary (55–112aaa; Young and Potter, 2002). The increase in plasma osmolality at 60aaa cannot be accounted for by increases in measured inorganic osmolytes (fall 200 mOsmol/kg short of the total osmolality). Although not measured, organic osmolytes such as neutral free amino acids (e.g., taurine and glycine) or small carbohydrates (e.g., myo-inositol) (Fiess et al., 2007) could possibly be an indicator of pathological tissue damage. In contrast to *P. lineatus*, very salinity tolerant species gradual increase plasma ion levels when acclimated to salinities up to about 70–75aaa, but thereafter then increase plasma ions in a linear fashion at higher salinities (see review by Gonzalez, 2012).

*Plotosus lineatus* challenged with BW, or hypoosmotic conditions are able to maintain plasma osmolality and Cl^-^ levels but not Na^+^. *Plotosus lineatus* were better able to regulate Cl^-^ levels than marine Ariid catfish, which do not have a DO and have higher serum Cl^-^ levels (Sulya et al., 1960; Pucke and Umminger, 1979). The lower plasma Na^+^ and hematocrit suggest a hemodilution but muscle water and ions were stable. Reports regarding the effect of lower salinity on MWC from different species vary from showing no effect (Woo and Chung, 1995; Kang et al., 2008) to increased MWC (Jensen et al., 1998; Kelly et al., 1999; Sinha et al., 2015).

Due to the dominance of the strong ions Na^+^ and Cl^-^ in blood, changes in the Na^+^/Cl^-^ ratio (SIR) has been recommended for indicating acid-base imbalances (Jensen et al., 1998; Sinha et al., 2015). In the present study, the direct measurements of plasma acid-base balance were not done due to the small size of the fish; however, calculations of SIR revealed changes in the plasma levels of weak anions (e.g., HCO_3_^-^) and thus acid-base balance. The BW SIR suggests a metabolic acidosis which has also been observed in European sea bass *D. labrax*, reared in lower salinity (Sinha et al., 2015). However, this contrasts with work by Jensen et al. (1998) who have reported a markedly increased plasma SIR following transfer to FW and slight decrease in HSW in *D. labrax*. In *P. lineatus*, HSW had no effect on SIR suggesting no alteration in acid-base status.

### Evidence for a Role of Gills in Salt Secretion?

The gill is typically linked to active ion regulation in teleost fishes (Evans, 2008). This is reflected in high levels of NKA, a central driver of ion transport, with dependency of the gill NKA to environmental salinity that may be altered by life history stage, species and experimental conditions in some cases (Varsamos et al., 2001; Evans, 2008; Malakpour Kolbadinezhad et al., 2012). However, branchial NKA activity of *P. lineatus* was the lowest of the osmoregulatory organs tested, unresponsive to HSW acclimation, and an order of magnitude lower than levels in the DO irrespective of salinity. A similar pattern has been reported in the sharks *Carcharhinus leucas* (Pillans et al., 2005) and *Chiloscyllium punctatum* (Cramp et al., 2015) and ray *Dasyatis sabina* (Piermarini and Evans, 2000) which possess the extra-branchial salt secreting organ the rectal gland. In elasmobranchs, the gills have a secondary function in ion regulation (Wilson et al., 2002; Evans, 2008). Our results confirm a similarity between gills of *P. lineatus* (Pucke and Umminger, 1979) and elasmobranchs underlining the potential role of DO in salt excretion (Van Lennep, 1968).

The IHC result of few branchial NKA-IR cells was consistent with NKA activity levels and in contrast to observations in most marine teleost fishes (e.g., alewife *Alosa pseudoharengus* Christensen et al., 2012; tilapia *Sarotherodon melanotheron* Ouattara et al., 2009). The few NKA-IR cells were restricted to the filament epithelium, leaving the lamella unimpeded for gas exchange (Evans, 2008; Henriksson et al., 2008). Also, it was very rare to find NKA-IR cells that co-expressed NKCC1, although apical CFTR staining was observed in NKA-IR cells. NKCC1 is a key component of the mechanism of secondary active Cl^-^ secretion and is abundantly expressed in seawater type gill ionocytes in teleost fishes (see review by Hiroi and McCormick, 2012). In elasmobranchs, NKCC1 mRNA expression has been detected in the gills of spiny dogfish *Squalus acanthias* (Xu et al., 1994); however, in the branchial epithelium of *C. punctatum* NKCC1 could not be immunolocalized (Cramp et al., 2015). This contrasts with the freshwater stingray *Himantura signifer* where NKCC1 is co-expressed in gill NKA-IR cells following BW (20aaa) acclimation (Ip et al., 2013). However, the rectal gland is absent in this species. The observation of ovoid cells deep within the filament epithelium which show only NKCC1-IR at HSW are unusual and their potential role has not been determined.

Elasmobranch gills also possess a V-ATPase rich cell that is involved in acid base regulation (Wilson et al., 1997; Piermarini and Evans, 2001; Tresguerres et al., 2006). Based on our IHC results, this cell type also appears in *P. lineatus*, and under HSW conditions immunoblotting results indicated a higher expression level. In killifish, basolateral V-ATPase has also been found in ionocytes (Katoh et al., 2003). Thus if the gills of *P. lineatus* have taken on the primary role in acid-base regulation, these cells maybe involved, although their basal localization in the epithelium makes this less certain.

### Evidence for the Role of the Dendritic Organ in Salt Secretion?

The higher DO NKA specific activity relative to the other ion regulatory organs, notably the gills, strongly indicates a role for this organ in NaCl secretion. This has also been seen in elasmobranchs with higher rectal gland NKA specific activity compared to their gills (*D. sabina,* Piermarini and Evans, 2000; *C. leucas*, Pillans et al., 2005; *C. punctatum,* Cramp et al., 2015). It has been demonstrated in euryhaline elasmobranchs that rectal gland NKA specific activity is higher in SW compared to FW acclimated animals (Piermarini and Evans, 2000; Pillans et al., 2005), although not in response to a moderate HSW acclimation (40aaa; Cramp et al., 2015). In *P. lineatus,* DO NKA specific activity was also higher in SW versus BW (3aaa) acclimated fish, but unexpectedly was also lower in HSW compared to SW fish. We predicted a similar if not higher NKA specific activity (Cramp et al., 2015). However, when we took into consideration the DO mass which was higher in HSW so that the total DO NKA activity was also higher suggesting an increase in overall capacity. In contrast, *C. punctatum* acclimated to 40aaa did not alter rectal gland size (Cramp et al., 2015). However, larger rectal glands of *D. sabina* (Piermarini and Evans, 1998), *Pristis perotteti* (Gerzeli et al., 1976) and *C. leucas* (Gerzeli et al., 1969; Oguri, 1976) captured in SW compared to FW have been reported. Moreover, rectal glands of FW stenohaline elasmobranchs are small to vestigial (Thorson et al., 1978).

However, the apparent hypertrophy of the DO may not be adaptive but rather pathological (inflammation or similar) as supported by the dramatic Hsp70 increase. In either case, it is clear that *P. lineatus* are significantly challenged by HSW and that the DO may be of limited use under such extreme conditions. Observation of a slightly albeit significantly larger DO in BW compared to the SW control fish suggests a high capacity of *P. lineatus* to move easily between different salinities, however, this was not sufficient to increase DO total NKA activity. Since the tissue sampling for the NKA activity measurement had been done after 14 days of acclimation, time course sampling would be necessary to have a comprehensive view of NKA activity in the DO of *P. lineatus*.

Strong basolateral tubular system immunoreactivity of NKA and NKCC1 in parenchymal cells of the DO indicates an ion secretory role in hypo-osmoregulation. The basolateral distribution of NKA and NKCC1 in other vertebrate salt secreting organs has also been demonstrated (Lytle et al., 1995; Wilson et al., 2002; Evans, 2008; Babonis et al., 2009; Babonis and Evans, 2011). Immunoblot results for NKA α subunit and NKCC (T4) were consistent in molecular mass compared to other vertebrates (Lytle et al., 1995; Blanco and Mercer, 1998, respectively). Finding multiple bands of NKCC might be the result of higher NKCC1 expression and immunoreactivity with either NKCC2 or NCC, reported in different species (Lorin-Nebel et al., 2006; Hiroi et al., 2008; Inokuchi et al., 2008; Christensen et al., 2012; Chew et al., 2015). Alternatively, the lipophilic nature of the NKCC migration through SDS-PAGE gels for immunoblotting analysis, or possibly the glycosylated monomer variability and/or different degrees of glycosylation could explain the banding patterns observed (Pelis et al., 2001; Tipsmark et al., 2002; Christensen et al., 2012; Chew et al., 2015). In BW, detection of NKCC suggests that maintaining a proportion of active NKCC for acid-base and/or cell volume regulation is important (Gamba, 2005) or it may be present as an inactive non-phosphorylated pool to be quickly activated for an acute response to higher salinity (Flemmer et al., 2010; Christensen et al., 2012). Regarding the expected increase of salt loading as a result of increased drinking and passive uptake under HSW conditions (see review by Grosell, 2010; Gonzalez, 2012), we detected significantly higher protein expression of DO NKCC representing an adaptation to increased salt excretion capacity.

IHC results for CFTR may reveal the possibility of a different isoform, which cannot be consistently recognized by the monoclonal antibody which is raised against a specific epitope of CFTR (Li et al., 2014). Pucke and Umminger (1979) detected an accumulation of Cl^-^ ions in the DO epithelium and proposed it was functional in salt secretion. The presence of CFTR in salt glands of birds, elasmobranchs and reptiles has been confirmed (Shuttleworth and Hildebrandt, 1999) although the antibody used in the present study does not show crossreactivity with elasmobranch (J. M. Wilson, personal observations), or sea snake (Babonis and Evans, 2011) salt glands or salmonid (S. D. McCormick, personal observations) gill CFTRs. Although *cftr* transcript was detected in the DO, predicted salinity dependent expression differences were not observed. Obviously, identifying the putative apical Cl^-^ channel in *P. lineatus* DO in future work would firmly establish the presence of the typical ion secretory cell of vertebrate salt glands.

The inconsistent results between NKA activity, α subunit protein levels and *atp1a1* mRNA expression levels may be related to post-transcriptional, or post-translational processing, phosphorylation state or modulation of the NKA kinetic properties by FXYD proteins interactions, stress or failing physiology (McDonough et al., 1990; Garty and Karlish, 2006; Tipsmark, 2008; Wang et al., 2008; Hauck et al., 2009; Tipsmark et al., 2010; Reilly et al., 2011; Christensen et al., 2012). Further investigation would be necessary to determine the effect of different salinities (FW to hypersaline) on various isoforms of NKA, their mRNA abundances and likely changes with salinity that would be helpful in interpreting the osmoregulatory function of the DO. The changes in *P. lineatus* PAT1 mRNA (*slc26a6a*) suggest the possible contribution of the DO to acid-base regulation in *P. lineatus*, although there was a lack of changes in V-ATPase and Ca17 protein expression.

### Role of the Kidney

Kidney NKA activity is typically responsive to environmental salinity (e.g., Venturini et al., 1992; Kelly and Woo, 1999; Herrera et al., 2009; Tang et al., 2012) although in some species no changes were observed (Sangiao-Alvarellos et al., 2005; Fuentes et al., 2006; Arjona et al., 2007). In the case of *P. lineatus* kidney NKA activity there was a positive relationship with environmental salinity, whereas in many euryhaline fishes the opposite was observed (Madsen et al., 1994; Kelly and Woo, 1999; Lin et al., 2004; Nebel et al., 2005; Tang et al., 2012; Yang et al., 2016). This higher NKA activity at lower salinities has been associated with increased urine production and the need to increase ion reabsorption which is driven by NKA (McDonald, 2007) whereas in the case of *P. lineatus* and a few other marine teleosts (Deane and Woo, 2004; Herrera et al., 2009; Yang et al., 2016) the increased NKA activity could potentially augment active ion secretion. In the case of the Plotosidae catfish *C. macrocephalus* hyperosmotic urine production has been reported (Kowarsky, 1973) although reports in other teleost species have been sporadic (*Fundulus kansae* Fleming and Stanley, 1965; *Paralichthys lethostigma* Hickman, 1968; *Opsanus beta* McDonald and Grosell, 2006) but are well worth investigating further.

In BW higher mRNA expression of *cftr* and *slc26a6a* suggest a functional role of the *P. lineatus* kidney in a regulatory role in Cl^-^ and HCO_3_^-^ transport that may be important for addressing the acid-base disturbance indicated by the lower strong ion difference (Jensen et al., 1998). However, based on a lack of changes in either Ca17 or V-ATPase protein expression, we cannot confirm their involvement.

### Role of the Intestine

The gastrointestinal tract of marine teleost is involved in osmoregulation through desalination of the imbibed seawater in the esophagus accompanied by NaCl coupled water uptake (see review by Grosell, 2010; Whittamore, 2012). Drinking rates were not measured in this study although there is ample evidence that shows a positive correlation with salinity (Whittamore, 2012). The anterior intestine has higher NKA activity than the posterior intestine but it is only the posterior intestine that is responsive to HSW. In Gulf toadfish *O. beta* higher NKA activity in anterior than posterior intestine was also observed (Guffey et al., 2011). Ruiz-Jarabo et al. (2016) have also found significant increases of NKA activity in the posterior region rather than the anterior in common galaxias *Galaxias maculates* in response to HSW. The intestine has been shown to respond to the increased drinking rate by increasing intestine NKA activity and expression, in addition to a number of key transporters and/or enzymes to coupled water absorption by the intestinal epithelium as reported in different species (see review by Grosell, 2010; Whittamore, 2012). There are also reports of variation between the anterior or posterior intestines in water absorption and/or ion secretion in marine teleosts (Aoki et al., 2003; Kim et al., 2008; Raldúa et al., 2008; Grosell, 2010; Gregório et al., 2013; Madsen et al., 2014).

Ion absorption via NKCC2/NCC might be occurring in all intestinal regions as demonstrated by IHC and in agreement with a number of other studies (e.g., Kalujnaia et al., 2007; Wilson and Castro, 2010; Esbaugh and Cutler, 2016). It has been demonstrated that because of reduced luminal Na^+^, and, therefore, Cl^-^ concentration along the intestine from anterior to posterior (Marshall and Grosell, 2006), the cotransport function of NKCC2 may be limited so would be more reliant on Cl^-^/HCO_3_^-^ exchange to aid water reabsorption through alkalinization of the gut and divalent cation precipitation (Grosell et al., 2009a; Grosell, 2010; Taylor et al., 2010). In support, observations were made of yellow-whitish precipitates, particularly in the posterior intestine of fish acclimated either in SW or HSW presumably made of Ca^2+^ and Mg^2+^ carbonates (Grosell, 2010; Madsen et al., 2014). The precipitation of carbonates decreases the osmotic gradient supporting water absorption (see review by Grosell, 2010; Whittamore, 2012). Finding absolute rates of water absorption in the anterior and posterior intestines of *P. lineatus* acclimated to different salinities would help address the relative roles of the two regions to water absorption.

CFTR localization to the apical membrane of spindle shaped columnar cells may be responsibile for ion (and fluid) secretion as has been reported in Atlantic killifish (Marshall et al., 2002). However, there are some other studies that did not observe ion or fluid secretion by the intestine (Field et al., 1980; Loretz, 1995). In sea bream *S. aurata* the apical region of the anterior intestinal epithelium showed a diffuse staining pattern of CFTR while it was more in the rectum of high salinity fish (Gregório et al., 2013). The observation of higher mRNA expression of *cftr* in the anterior intestine at HSW suggests a role in the recycling Cl^-^ in parallel with the apical Cl^-^/HCO_3_^-^ exchanger to increase HCO_3_^-^ excretion (Grosell and Taylor, 2007; Taylor et al., 2010). The presumed Cl^-^/HCO_3_^-^ exchanger is possibly *slc26a6a* (PAT1). Its mRNA expression was detected in intestine but it was not responsive to salinity.

The localization of V-ATPase in the intestine was generally more pronounced in the anterior than the posterior intestine regardless of acclimation salinity. In Gulf toad fish acclimated in either SW or HSW, V-ATPase showed apical and basolateral localization in enterocyte (Guffey et al., 2011). The apical localization of V-ATPase in fish enterocytes has been proposed to aid in carbonate precipitation to maintain water absorption rates in marine fishes (Grosell et al., 2009a,b; Guffey et al., 2011; Gregório et al., 2013).

### Cellular Stress and Salinity

The heat shock proteins (Hsps) are expressed in cells and are involved in maintaining a number of vital cellular processes as part of the cellular stress response (Hightower, 1991; Morimoto and Santoro, 1998; Basu et al., 2002; Iwama et al., 2006). Deane and Woo (2004, 2011) have shown that salinity can induce a cellular stress response. In the DO and kidney Hsp70 levels are highest at HSW indicating a cellular stress requiring the activation of stress protein mechanisms to provide protective actions against stress situations [for more details see review by Deane and Woo (2011)]. Elevated intracellular ion concentrations typically can be correlates with intracellular damage (Burg et al., 2007). However, given the lack of differences in Hsp70 levels in the gills, or intestine at salinity extremes suggests less of a stress compare to the DO and kidney or a different threshold of salt tolerance. In juvenile sharks challenged with HSW (41aaa), gill Hsp70 did not change in *Mustelus antarcticus* but did in *Galeorhinus galeus* (Tunnah et al., 2016).

### Summary

**FIGURE 8:**
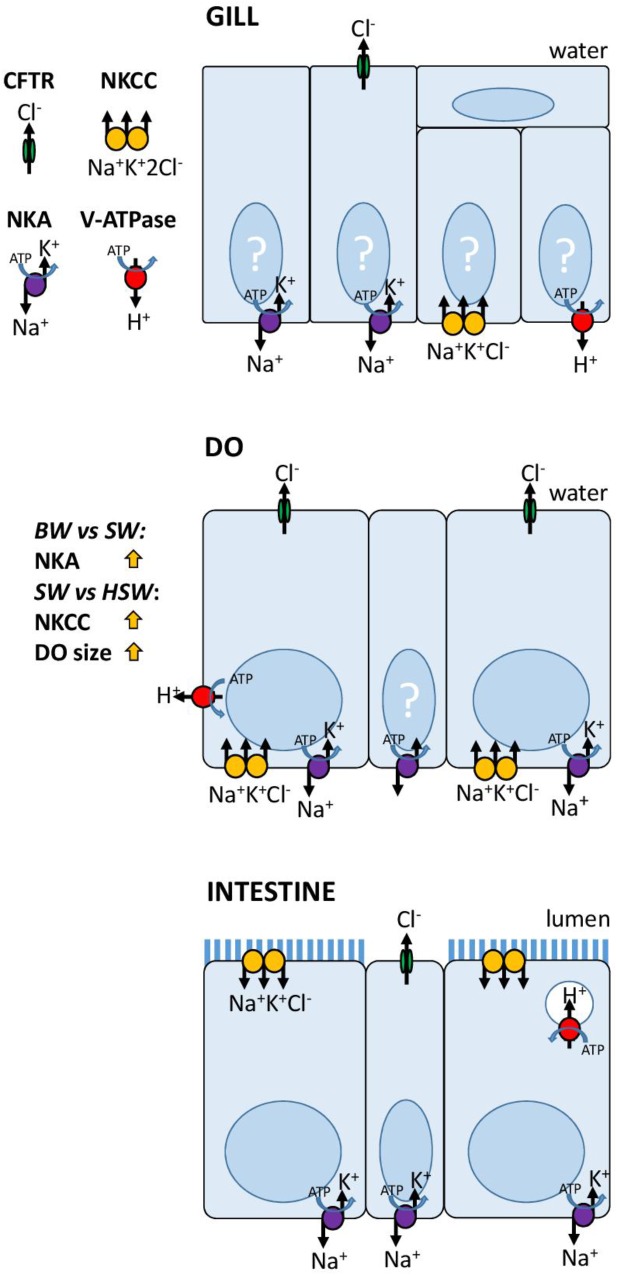
Models for ion transport mechanisms in *P. lineatus* gill, DO and intestine.

In summary, the salt secreting function of the DO has been proposed based on physiological (Kowarsky, 1973), ecological (Lanzing, 1967), and ultrastructural (Van Lennep, 1968) evidence. Our molecular observations summarized in **Figure 8**, show a strikingly high NKA activity, and localization of NKA, NKCC1 and CFTR in the DO consistent with the hypothesis suggesting a conservation of the mechanism of ion transport in the secretory cells of vertebrate salt secreting organs (Babonis and Evans, 2011). The gills of *P. lineatus* are unlike those of other marine teleosts and more similar to the gills of elasmobranch fishes in terms of their significances to ion regulation. The *P. lineatus* kidney is particularly responsive to salinity and requires further study. The unique osmoregulatory strategy of the Plotosidae catfishes amongst the teleosts can be linked to their independent invasion of the marine environment by a freshwater siluriform ancestor (Lanzing, 1967; de Pinna, 2005). However, the breakdown of osmoregulatory homeostasis under HSW conditions indicated that this strategy is of limited use under more extreme salinity conditions.

## Author Contributions

JMW and SMK designed the experiments. SMK performed the experiments, analyzed the data, and wrote the draft of the manuscript. JC and JMW were also involved in the analysis of data and writing and editing of the manuscript.

## Conflict of Interest Statement

The authors declare that the research was conducted in the absence of any commercial or financial relationships that could be construed as a potential conflict of interest.

## Supplementary Material

FIGURE S1 Immunoblotting tissue profile from *P. lineatus* Cftr (160 kDa), NKCC (240 kDa), NKA (100 kDa), V-ATPase (56 kDa) and Ca17 (30 kDa). Bars (left) indicate bands of interest. Gi, Gill; Kd, Kidney; DO, Dendritic organ; AI, Anterior Intestine; PI, Posterior Intestine; Rc, Rectum; Br, Brain; Li, Liver; Mu, Muscle.

FIGURE S2 Na^+^/K^+^-ATPase α-subunit (Atp1a) phylogenetic tree. The evolutionary history was inferred using the Neighbor-Joining method. The optimal tree with the sum of branch length = 0.85546745 is shown. The percentage of replicate trees in which the associated taxa clustered together in the bootstrap test (1000 replicates) are shown next to the branches. The tree is drawn to scale, with branch lengths in the same units as those of the evolutionary distances used to infer the phylogenetic tree. The evolutionary distances were computed using the Poisson correction method and are in the units of the number of amino acid substitutions per site. The analysis involved 11 amino acid sequences. All positions containing gaps and missing data were eliminated. There were a total of 198 positions in the final dataset. Evolutionary analyses were conducted in MEGA7.

TABLE S1 Nucleotide sequences and amplicon sizes of primers used in the present study for RT-PCR and qPCR (*actb*, β-actin; *atp1a1*, Na^+^/K^+^-ATPase; *cftr*, Cystic fibrosis transmembrane conductance regulator; *ca17*, Cytosolic carbonic anhydrase; *slc26a6*, Putative Anion Transporter Cl^-^/HCO_3_^-^ exchanger gene.

TABLE S2 **(A)** RT-PCR and **(B)** qPCR profiles (*actb*, β-actin; *atp1a1*, Na^+^/K^+^-ATPase; *cftr*, Cystic fibrosis transmembrane conductance regulator; *ca17*, Cytosolic carbonic anhydrase; *slc26a6*, Putative Anion Transporter Cl^-^/HCO_3_^-^ exchanger gene).

TABLE S3 Comparisons of the amino acid sequence identities of *Plotosus lineatus* Atp1a1, Cftr, Ca17 and Slc26a6 partial sequences with respective orthologues from channel catfish (*I. punctatus*), zebrafish (*D. rerio*) and rainbow trout (*O. mykiss*).
