# Supplementary figures and images for: Osmoregulation in the Plotosidae Catfish: Role of the Salt Secreting Dendritic Organ
Source: Front Physiol. 2018 Jul 3;9:761. doi: 10.3389/fphys.2018.00761 (PMC6037869; doi:10.3389/fphys.2018.00761)

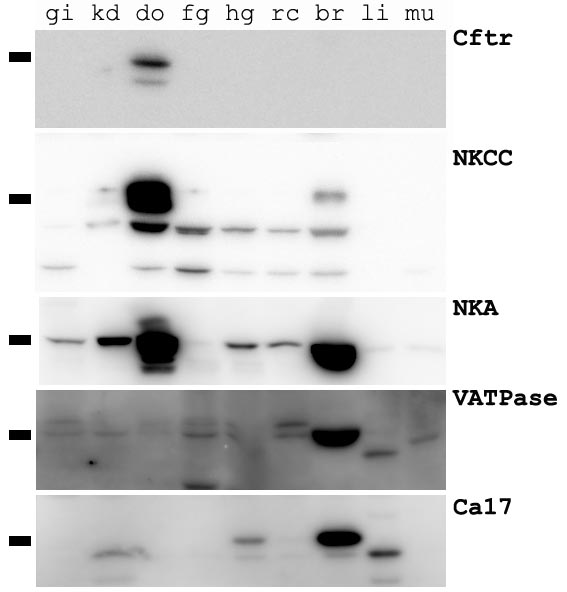

Supplement: FIGURE S1 — Immunoblotting tissue profile from P. lineatus Cftr (160 kDa), NKCC (240 kDa), NKA (100 kDa), V-ATPase (56 kDa) and Ca17 (30 kDa). Bars (left) indicate bands of interest. Gi, Gill; Kd, Kidney; DO, Dendritic organ; AI, Anterior Intestine; PI, Posterior Intestine; Rc, Rectum; Br, Brain; Li, Liver; Mu, Muscle. [file Image_1.JPEG]

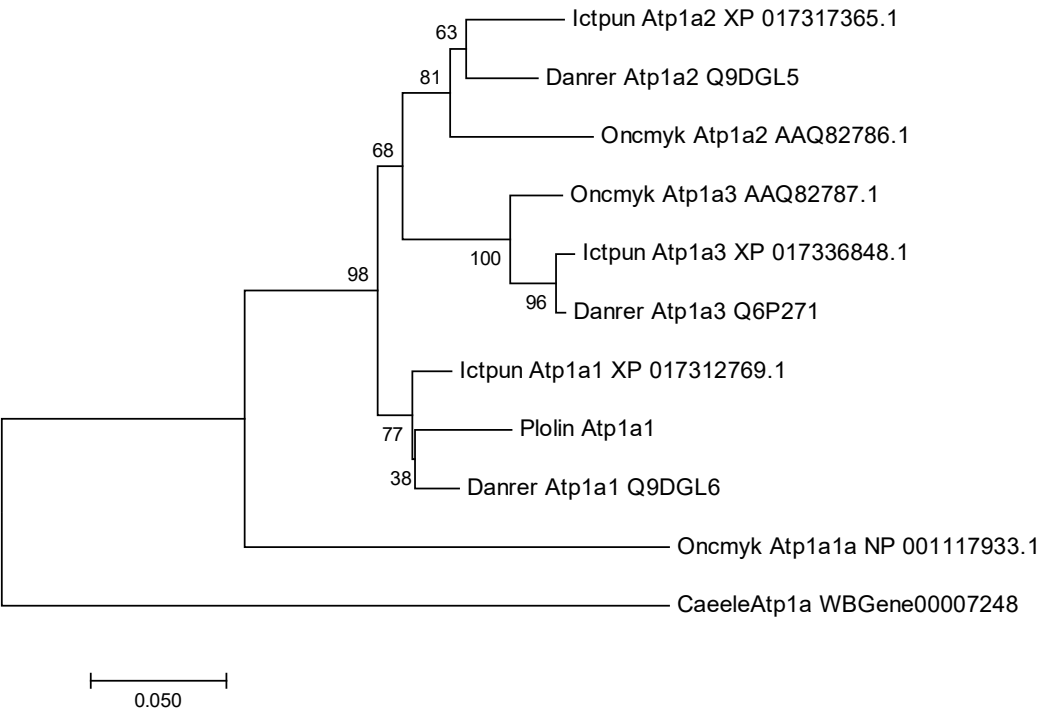

Fig S2

Supplement: FIGURE S2 — Na+/K+-ATPase α-subunit (Atp1a) phylogenetic tree. The evolutionary history was inferred using the Neighbor-Joining method. The optimal tree with the sum of branch length = 0.85546745 is shown. The percentage of replicate trees in which the associated taxa clustered together in the bootstrap test (1000 replicates) are shown next to the branches. The tree is drawn to scale, with branch lengths in the same units as those of the evolutionary distances used to infer the phylogenetic tree. The evolutionary distances were computed using the Poisson correction method and are in the units of the number of amino acid substitutions per site. The analysis involved 11 amino acid sequences. All positions containing gaps and missing data were eliminated. There were a total of 198 positions in the final dataset. Evolutionary analyses were conducted in MEGA7. [file Image_2.pdf]
