# Supplementary material for: Osmoregulation in the Plotosidae Catfish: Role of the Salt Secreting Dendritic Organ
Source: Front Physiol. 2018 Jul 3;9:761. doi: 10.3389/fphys.2018.00761 (PMC6037869; doi:10.3389/fphys.2018.00761)
Supplement: TABLE S1 — Nucleotide sequences and amplicon sizes of primers used in the present study for RT-PCR and qPCR (actb, β-actin; atp1a1, Na+/K+-ATPase; cftr, Cystic fibrosis transmembrane conductance regulator; ca17, Cytosolic carbonic anhydrase; slc26a6, Putative Anion Transporter Cl-/HCO3- exchanger gene. [file Table_1.pdf]

**Table S1.** Nucleotide sequences and amplicon sizes of primers used in the present study for RT-PCR and qPCR (*actb*, β-Actin; *atp1a1*, $Na^+/K^+$-ATPase; *cftr*, Cystic fibrosis transmembrane conductance regulator; *ca17*, Cytosolic carbonic anhydrase; *slc26a6*, Putative Anion Transporter $Cl^-/HCO_3^-$ exchanger gene.

| | RT-PCR | | qPCR | |
|---|---|---|---|---|
| **Primer** | **Sequence** (5′-3′) | **Size** (bp) | **Sequence** (5′-3′) | **Size** (bp) |
| ***actb*** | **Fw-**GGCCGCGACCTACAGACTAC<br>**Rev-**ACCGAGGAAGGATGGCTGGAA | 250 | **Fw-**CCCTCGTGCTGTGTTCCCATC<br>**Rev-**CTCTTGCTCTGTGCCTCATCTCC | 108 |
| ***atp1a*** | **Fw-** GGTATCGCTGGGTCTGATGT<br>**Rev-** AGGATGGAGTTCCTCCTGGT | 632 | **Fw-** CCACTTTAGCCTCTCTGATGAC<br>**Rev-**ATAACCTTGATTCCAGCACTCC | 183 |
| ***cftr*** | **Fw-** TTTTCTGGTGGACAAGCCCT<br>**Rev-** ATCGGGAAAGTGCTCGTAGG | 188 | **Fw-** TTTTCTGGTGGACAAGCCCT<br>**Rev-** ATCGGGAAAGTGCTCGTAGG | 188 |
| ***ca17*** | **Fw-** CAGTTCCATTTCCATTGGGG<br>**Rev-** CAGAGGAGGGGTGGTCAG | 333 | **Fw-** GGGGATCCAGTAATGAGAAGG<br>**Rev**- CAAGAAGACCCCAACCACAG | 152 |
| ***slc26a6*** | **Fw-** TGGTGCGGTTTGGATTTGTG<br>**Rev-** ACCAGTTCCTGGTTGCTGTC | 544 | **Fw-** ATCAATCCGAACCGCTACAG<br>**Rev-** AACAGGAATCGGAACAGGAA | 183 |

**Table S2.** (**A**) RT-PCR and (**B**) qPCR profiles (*actb*, β-Actin; *atp1a1*, $Na^+/K^+$-ATPase; *cftr*, Cystic fibrosis transmembrane conductance regulator; *ca17*, Cytosolic carbonic anhydrase; *slc26a6*, Putative Anion Transporter $Cl^-/HCO_3^-$ exchanger gene).

**A**

| Step | RT-PCR | *actb* | *atp1a* | *cftr* | *ca17* | *slc26a6* |
|---|---|---|---|---|---|---|
| 1 | Denaturation hot start | 95°C 2min | 98°C 10s | 98°C 10s | 98°C 10s | 98°C 10s |
| 2 | Denature | 95°C 30s | 98°C 1s | 98°C 1s | 98°C 1s | 98°C 1s |
| 3 | Anneal | 60°C 30s | 60°C 5s | 58°C 5s | 56°C 5s | 58°C 5s |
| 4 | Extend | 72°C 5s | 72°C 5s | 72°C 5s | 72°C 3s | 72°C 3s |
| 5 | Repeat 2-4 | 30 | 35 | 35 | 35 | 35 |
| 6 | Final Extend | 72°C 5min | 72°C 2min | 72°C 2min | 72°C 2min | 72°C 2min |

**B**

| qPCR Step | *actb* | *atp1a* | *cftr* | *ca17* | *slc26a6* |
|---|---|---|---|---|---|
| Denaturation and hot start | 95°C 3min | 95°C 3min | 95°C 3min | 95°C 3min | 95°C 3min |
| Denaturation | 95°C 10s | 95°C 10s | 95°C 10s | 95°C 10s | 95°C 10s |
| Annealing | 60°C 30s | 60°C 30s | 58°C 30s | 56°C 30s | 58°C 30s |
| Extension | 72°C 5s | 72°C 3s | 72°C 5s | 72°C 3s | 72°C 3s |
| Cycle # | 30 | 35 | 35 | 35 | 35 |

**Table S3.** Comparisons of the amino acid sequence identities of *Plotosus lineatus* Atp1a1, Cftr, Ca17 and Slc26a6 partial sequences with respective orthologues from channel catfish (*I. punctatus*), zebrafish (*D.rerio*) and rainbow trout (*O.mykiss*).

| | Atp1a1 (198 aa) | | Cftr (43 aa) | | Ca17 (111 aa) | | Slc26a6 (166 aa) | |
|---|---|---|---|---|---|---|---|---|
| *I. punctatus* | 0.949 | XP_017312769.1 | 0.883 | XP_017321934.1 | 0.855 | XP_017322776.1 | 0.801 | XP_017323671.1 |
| *D. rerio* | 0.949 | Q9DGL6 | 0.813 | NP_001038348.1 | 0.747 | F1R454 | 0.710 | XP_001344243.4 |
| *O. Mykiss* | 0.924 | Q6VYM6 | 0.720 | XP_021432274.1 | 0.756 | Q6R4A2 | 0.536 | XP_021422617.1 |
